# Suppression of exercise-induced neutrophilia and lymphopenia in athletes by cystine/theanine intake: a randomized, double-blind, placebo-controlled trial

**DOI:** 10.1186/1550-2783-7-23

**Published:** 2010-06-04

**Authors:** Shigeki Murakami, Shigekazu Kurihara, C Alan Titchenal, Masaru Ohtani

**Affiliations:** 1 Department of Sports Science, School of Health and Sports Science, Juntendo University, Chiba, Japan; 2 Research Institute for Health Fundamentals, Ajinomoto Co., Inc., Kanagawa, Japan; 3 University of Hawaii at Manoa, Honolulu, HI, USA; 4 Department of Frontier Sciences, The University of Tokyo, Chiba, Japan

## Abstract

**Background:**

Intense exercise induces increased blood neutrophil counts and decreased lymphocyte counts, and leads to inflammation and immunosuppression. It was previously reported that cystine and theanine (CT) supplementation by long-distance runners before a training camp suppressed the changes of these blood parameters observed in un-supplemented control subjects after the camp. The purpose of the present study was to determine the effects of CT supplementation on the inflammatory response and immune state before and after intense endurance exercise in long-distance runners at a training camp.

**Methods:**

Sixteen long-distance runners were allocated to one of two groups given CT supplements (700 mg cystine + 280 mg theanine daily) or placebo (8 in each group) for 7 days prior to and during a 9-day training camp. Daily run training averaged 19.9 km/day prior to the camp and 28.6 km/day during the camp. On the initial and final days of the camp, blood samples were collected before and after 15 km morning interval running workouts (1000 m × 15 times) and analyzed for neutrophil and lymphocyte counts and myoglobin.

**Results:**

The relative change in exercise-induced blood neutrophil count (% of pre-exercise values) was significantly lower in the CT group than in the placebo group (163.3 ± 43.2% *vs. *200.4 ± 19.6%, p = 0.044) on the initial day of camp, but not on the last day. The decline in lymphocyte count (% of pre-exercise values) was significantly less in the CT group than in the placebo group (60.2 ± 19.2% *vs. *36.2 ± 12.0%, p = 0.010) on the initial day of camp, but not on the last day. In blood myoglobin, there was a trend toward lower % of pre-exercise values in the CT group (p < 0.09) on both measurement days.

**Conclusion:**

CT supplementation significantly attenuated the increase in neutrophil count and the reduction in lymphocyte count induced by intense endurance exercise. These results suggest that CT supplementation may suppress the exercise-induced fluctuation of the blood immunocompetent cells and may help to reduce the alteration of the immune state.

## Background

Cystine, a dipeptide of the sulfur amino acid cysteine, is a precursor of glutathione (GSH) that is responsible for the antioxidant response in the body, and its supply is limiting in the synthesis of GSH[1]. On the other hand, theanine is an amino acid abundant in green tea and is known to be metabolized to glutamic acid and ethylamine within the intestinal tract, liver, *etc. *[2,3]. A recent experiment in mice indicated that oral administration of cystine and theanine (CT) reinforces GSH synthesis and humoral immune responses after antigen stimulation, and, as a result, reinforces antigen-specific antibody production [4]. In this report, CT increased the levels of total GSH and the serum IL-10/IFN-γ ratio related to the balance of T helper (Th) 1/Th2 cell responses after immunization. As a result, CT enhanced serum antigen-specific IgG production via the increased Th2-mediated responses after immunization [4]. In the analysis on the model of influenza virus infection using aged mice, CT also was reported to decrease the lung viral titer after infection through the increase of serum IL-10/IFN-γ ratio and GSH synthesis in the spleen [5]. In addition, in a clinical study in humans, Miyagawa *et al. *reported that oral administration of CT improves antibody production in the elderly with lowered immune function at the time of flu vaccination [6].

It has been reported that athletes often experience overtraining syndromes where they are unable to sufficiently recover their physical condition after a certain period of intense, strenuous exercise [7,8]. This is due to lowered immunity, increasing the susceptibility to infectious disease (diarrhea, fever, pharyngitis, and symptoms of the common cold, *etc.*) during a prolonged period of fatigue and reduced physical performance [8,9]. With regard to the potential mechanisms underlying this phenomenon, it has been reported that such prolonged periods of intense endurance exercise are accompanied by increases in inflammatory cytokine concentrations causing an immunosuppressive effect [10,11]. This immunosuppressive effect also has been reported to cause athletes to be more susceptible to infectious diseases of the respiratory system due to virus infection after intense exercise [12-15]. Recently, we reported that CT ingestion by long-distance runners before a training camp suppressed the increase in blood neutrophil counts and the decrease in lymphocyte counts observed in control subjects after the camp [16]. Similar to cysteine contained in CT, *N*-acethylcysteine (NAC), a precursor of GSH, was shown in clinical studies to significantly suppress reactive oxygen species (ROS) from neutrophils increased through exercise [17-19]. These findings suggested that CT ingestion may suppress the excessive inflammatory response induced by the accumulation of daily intense exercise and inhibit inflammatory-mediated immunosuppression and associated muscle damage in athletes. However, it is not clear whether CT ingestion can influence the above blood parameters before and after single bouts of intense exercise.

In the present study, we analyzed the effects of CT ingestion on the inflammatory response, immune state, and indicators of muscle disruption before and after intense endurance exercise consisting of 15 km interval running workouts (1000 m × 15 times), in long-distance runners at a training camp.

## Methods

### Procedures

This experiment was performed in accordance with the principles of the Declaration of Helsinki and with the approval of the institutional review board (IRB) of Juntendo University School of Health & Sports Science as a randomized, double-blind, placebo-controlled, parallel-group study.

### Subjects

The subjects were 16 male long-distance runners (members of the Takaoka University of Law Track and Field team) attending a winter training camp as previously reported [16]. All subjects signed voluntary informed consent forms and received a detailed explanation regarding the procedures of the study. The 16 subjects were distributed evenly between the two groups considering their age and personal best time for the 5000 m run. Eight subjects were assigned to the cystine/theanine (CT) group and 8 subjects were assigned to the placebo (P) control group. The characteristics of the subjects in each group are presented in Table 1. The subjects all stayed in a dorm close to the campus and lifestyle, including meals and exercise before and during the training camp, was the same for all subjects. Based on food consumed, energy and nutrient intakes were as follows (mean ± SE): energy: 2144 ± 81 kcal, protein: 80.4 ± 4.8 g, fat: 49.8 ± 5.9 g, carbohydrate: 329.6 ± 13.7 g, calcium: 340.4 ± 59.8 mg, socium chloride: 13.2 ± 0.9 g.

**Table 1:** Subject characteristics.

		P group (n = 8)	CT group (n = 8)
	Age (year)	20.0 ± 0.9	20.0 ± 0.9
	Height (cm)	170.9 ± 5.0	171.0 ± 6.8
	Weight (kg)	55.8 ± 3.9	56.5 ± 5.0
	Personal best time for 5000 m run	15 min 5 s ± 23 s	15 min 9 s ± 24 s

Values are means ± SEM.

### Dosage and method

Following the methodology used previously in a clinical study in humans by Miyagawa *et al. *[6] and in our previous study [16], the active ingredients in CT consisted of 700 mg of cystine and 280 mg of theanine per pack (per day) in a granular form. P was also in granular form and contained 930 mg of crystalline cellulose and 50 mg of monosodium glutamate. In previous human trials of CT supplementation, CT was supplemented for 14 days before Flu vaccination [6], seven days before high-intensity resistance exercise [20] and 10 days before the endurance training camp in our previous study [16]. All of these trials reported starting CT supplementation at least 7 days before the vaccination or exercise stress. In the present trial, the period of CT supplementation was 8 days before the training camp and 8 days during the camp. The subjects ingested CT or P by the double-blind method from 7-22 February 2008 (16 days) after dinner every day before and during the winter training camp. The compliance rate of the ingestion was checked by collecting the empty pouches that had contained CT and P shortly after ingestion. The subjects were prohibited from taking green tea, other amino acids, proteins, or creatine 5 days before the start date until the end of the study. Also, these athletes generally did not take any supplements, such as amino acids, proteins and creatine.

### Amount of exercise

The 16 subjects took part in practice sessions at the track team practice field of Takaoka University of Law for 8 days from 7-14 February 2008, and at the winter training camp in Takamatsu, Kagawa prefecture, Japan, for 8 days from 15-22 February 2008; all 16 subjects participated in the same training programs during each of the two time periods. The average distance run by the subjects during the 8 days before the training camp was 19.9 km/day (mean of 4 days of training) compared to 28.6 km/day (mean of 7 days of training) during the 8 days of training camp. The training program before and during the training camp is summarized in Table 2.

**Table 2:** Summary of the training program before and during the training camp.

	Date		Training program
Before the training camp	8-Feb	Afternoon	5000-m interval runs × 3
	9-Feb	Afternoon	120-min run
	11-Feb	Afternoon	27-km cross-country run
	12-Feb	Afternoon	1500-m interval runs × 10

During the trainig camp	15-Feb	Morning	1000-m interval runs × 15
		Afternoon	20-km run
	16-Feb	Morning	15-km run
		Afternoon	30-km run
	17-Feb	Morning	15-km run
		Afternoon	2000-m interval runs × 7
	18-Feb	Afternoon	34-km run
	20-Feb	Afternoon	20-km run
	21-Feb	Morning	30-km run
	22-Feb	Morning	1000-m interval runs × 15

Blood and saliva samples were collected before and after the 1000-m interval runs × 15 performed in the morning on 15 and 22 February 2008.

For the two training sessions with 1000 m interval runs × 15 that were performed on the first and the last days of the training camp on February 15 (the temperature and humidity were 2°C and 38%, respectively) and 22 (the temperature and humidity were 3°C and 35%, respectively) of 2008, 16 subjects were assigned to 3 teams (A-C) according to ability. The number of the subjects was 4 in team A, 6 in team B, and 6 in team C and each team included the same number of CT or P group. Each 1000 m interval run was followed by a 200 m jog. Team A ran 1000 m in 3 min 15 s × 5, 3 min 10 s × 5, 3 min 5 s × 4, and then ran the last 1000 m interval at full speed (average run time: 3 min 5 s). Team B ran 1000 m in 3 min 20 s × 5, 3 min 15 s × 5, 3 min 10 s × 4, and then ran the last one at full speed (average run time: 3 min 9 s). Team C ran 1000 m in 3 min 25 s × 5, 3 min 20 s × 5, 3 min 15 s × 4, and then ran the last one at full speed (average run time: 3 min 16 s). The interval runs were performed so that the load of exercise was comparable regardless of the runners' abilities.

### Test schedule and analysis items

Blood and saliva samples were collected before and after the 1000-m interval runs × 15 performed in the early morning on 15 and 22 February 2008 on the first and last day of the training camp, respectively. The above samples were collected immediately after the subjects woke up in the early morning at 6 AM, before breakfast and before they engaged in any physical activities. After blood and saliva samples were collected, 1000-m interval runs × 15 training was performed from 7 AM, and blood and saliva samples were collected again after the training without any massage or pressure to the skeletal muscle. Nineteen ml of blood was collected from the antecubital vein by the standard procedure using a blood collection tube. White blood cell (WBC), neutrophil, and lymphocyte counts were measured using blood samples as part of a general peripheral blood test. In addition, blood levels of creatine phosphokinase (CPK), myoglobin (Mb) and IL-6 were included in the general biochemical examination and cortisol was measured in a saliva test. All analyses were performed in a biomedical clinical laboratory (Health Sciences Research Institute, Inc., Japan).

### Statistical analysis

Data are shown as the means ± SEM. The pre- and post-interval training data on the first and last days of the training camp for each group were compared for statistical significance using the paired *t*-test or Wilcoxon's signed rank test. Comparisons of relative changes between the groups in the data for blood and saliva samples at the time of collection were performed using the *t*-test or Mann-Whitney rank sum test. In addition, relative percentage changes in leukocyte, neutrophil, and lymphocyte counts as well as myoglobin levels before and after interval training were used to perform linear regression analysis. All statistical analyses were performed using SigmaStat3.1 software (Systat Software, Inc., Richmond, CA) and p < 0.05 was taken to indicate significance.

## Results

**Figure 1:**
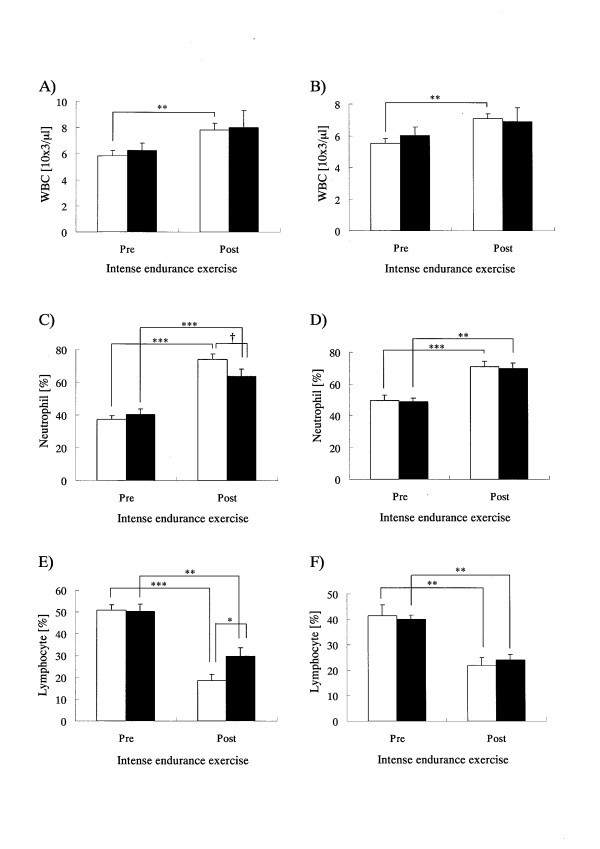
**Hematological parameters in the subjects pre- and post-intense endurance exercise on the initial (A, C, E) and final (B, D, F) days of the training camp**. Open and closed bars show the P and CT groups, respectively. Graphs A and B show mean levels of WBC counts, graphs C and D show mean levels of neutrophil counts and graphs E and F show mean levels of lymphocyte counts for pre- and post-intense endurance exercise. Values are means ± SEM. *, **, and *** Indicate significant difference (p < 0.05, p < 0.01, and p < 0.001, respectively).^† ^Indicates tendency for a difference (p < 0.1).

**Figure 2:**
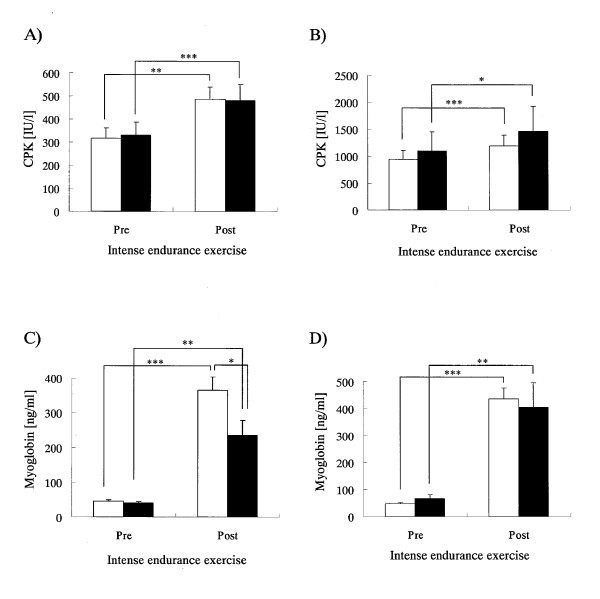
**Blood biochemical parameters in the subjects pre- and post-intense endurance exercise on the initial (A, C) and final (B, D) days of the training camp**. Open and closed bars show the P and CT groups, respectively. Graphs A and B show mean levels of CPK and graphs C and D show mean levels of Mb for pre- and post-intense endurance exercise. Values are means ± SEM. *, **, and *** Indicate significant difference (p < 0.05, p < 0.01, and p < 0.001, respectively).

**Figure 3:**
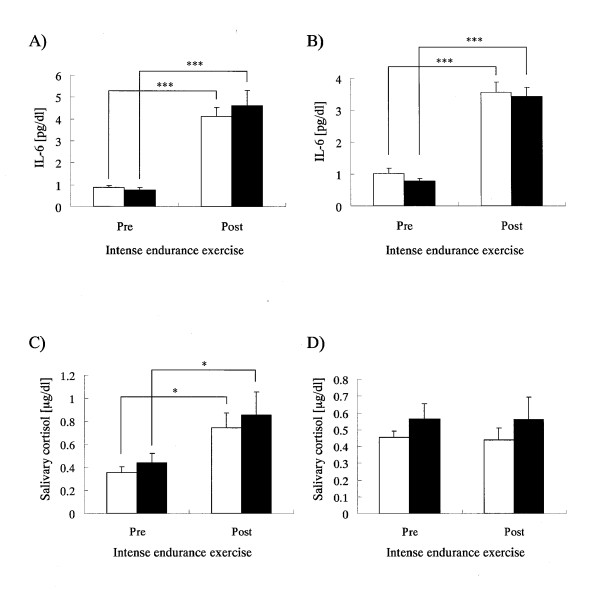
**Blood cytokine and salivary stress hormone levels in the subjects pre- and post-intense endurance exercise on the initial (A, C) and final (B, D) days of the training camp**. Open and closed bars show the P and CT groups, respectively. Graphs A and B show mean levels of blood IL-6 and graphs C and D show mean levels of salivary cortisol for pre- and post-intense endurance exercise. Values are means ± SEM. * and *** Indicate significant difference (p < 0.05 and p < 0.001, respectively).

As shown in Figure 1A, B) the blood WBC level in P group significantly increased after the interval training (1000-m interval runs × 15) on both the first and last days of the training camp, while no significant increase was observed in the CT group. No significant difference was observed in relative percentage increase of the WBC level accompanying the exercise on the first day of the training camp (Table 3), but for the last day of the training camp, the level in the CT group showed a lower trend compared to the P group (p = 0.083) (Table 3). The neutrophil count increased significantly in both groups after interval training on the first day of the training camp, and that in the CT group tended to be lower compared to the P group (p = 0.077) (Figure 1C). The relative percentage increase in neutrophil count on the first day of the training camp was significantly lower in the CT group compared to the P group, which indicated that the increase in the CT group was being suppressed (Table 3). The neutrophil count increased significantly in both groups after interval training on the last day of the training camp (Figure 1D), and there was no difference between the two groups in relative percentage increase (Table 3). The lymphocyte count decreased significantly in both groups after interval training on the first day of the training camp, and the value of the CT group was significantly higher than that of the P group (Figure 1E). The relative percentage reduction of lymphocyte count on the first day of the training camp was significantly lower in the CT group compared to the P group, indicating that the decrease was suppressed in the CT group (Table 3). Lymphocyte count decreased significantly after interval training on the last day of the training camp (Figure 1F), and there was no difference in relative percentage reduction between the two groups (Table 3). In addition, no significant change of blood hematocrit and hemoglobin concentration was observed between the pre- and post-interval training on the first and last days of the training camp in each group (data not shown). Blood CPK level increased significantly in both groups after interval training on both the first and last days of the training camp (Figure 2A, B), but there was no difference between the two groups in the relative percentage increase (Table 3). Blood Mb level increased significantly in both groups after interval training on the first day of the training camp, and the value in the CT group was significantly lower than that in the P group (Figure 2C). The relative percentage increase in Mb level on the first day of the training camp in CT group tended to be lower than that of the P group (p = 0.085), suggesting that the increase in the CT group was being suppressed (Table 3). Mb level increased significantly in both groups after interval training on the last day of the training camp (Figure 2D), and the relative percentage increase in the CT group tended to be lower than that of the P group (p = 0.083) (Table 3). Blood IL-6 level increased significantly in both groups after interval training on both the first and last days of the training camp (Figure 3A, B), but there was no difference between the two groups in the relative percentage increase (Table 3). Cortisol level in saliva increased significantly in both groups after interval training on the first day of the training camp (Figure 3C), but there was no difference in relative percentage increase between the two groups (Table 3). On the last day of the training camp, no increase was observed in the cortisol level in saliva in either group after interval training (Figure 3D), and there was no difference in relative percentage change between the two groups (Table 3).

**Table 3:** Post-intense endurance exercise blood values expressed as a percentage of the pre-intense endurance exercise values.

		P group (n = 8)	CT group (n = 8)	P value
Initial day of camp	WBC	136.7 ± 10.8	122.3 ± 11.6	0.381
	Neutrophil	200.4 ± 6.9	163.3 ± 15.3	0.044
	Lymphocyte	36.2 ± 4.2	60.2 ± 6.8	0.010
	CPK	157.7 ± 6.5	148.9 ± 5.9	0.335
	Myoglobin	823.6 ± 107.6	561.5 ± 92.0	0.085
	IL-6	514.4 ± 66.9	705.3 ± 117.0	0.279
	Coritisol	245.7 ± 52.3	185.9 ± 37.2	0.367

Final day of camp	WBC	129.5 ± 6.7	113.1 ± 7.5	0.083
	Neutrophil	149.5 ± 14.4	145.5 ± 10.0	0.824
	Lymphocyte	56.8 ± 9.5	61.2 ± 6.9	0.715
	CPK	128.1 ± 2.8	142.9 ± 10.6	0.130
	Myoglobin	936.6 ± 104.9	654.4 ± 143.3	0.083
	IL-6	406.3 ± 66.9	450.7 ± 41.1	0.581
	Coritisol	100.2 ± 17.8	102.1 ± 18.8	0.945

Percentage of pre-intense exercise values (means ± SEM).

To assess correlations among the percentage change of immunocompetent cell counts and Mb levels for each of the two interval training sessions, linear regression analysis was performed using relative percentage change before and after interval training (1000-m interval runs × 15) for all subjects (n = 16). As shown in Table 4, the relative percentage change of WBC on the first and last days of the training camp both tended to show positive correlations or significant positive correlations with percentage change of neutrophil count, and showed significant negative correlations with percentage change in lymphocyte count. In addition, the relative percentage change in neutrophil count on the first and last days of the training camp showed significant negative correlations with percentage change in lymphocyte count. Relative percentage change of neutrophil count on the first day of the training camp tended to show a positive correlation to the percentage change in Mb level, but this was not observed on the last day of the training camp. Relative percentage change in lymphocyte count on the first day of the training camp showed a significant negative correlation with the percentage change in Mb level; however, as seen with neutrophil count, this was not observed on the last day of the training camp.

**Table 4:** Associations among intense exercise-induced responses of immune cells and index for muscle damage.

	Dependent variable (n = 16)	Independent valiable (n = 16)	R value	P value
Initial day of camp	WBC	Neutrophil	0.455	0.076
	WBC	Lymphocyte	-0.517	0.040
	Neutrophil	Lymphocyte	-0.793	<0.001
	Neutrophil	Myoglobin	0.471	0.066
	Lymphocyte	Myoglobin	-0.690	0.003

Final day of camp	WBC	Neutrophil	0.517	0.040
	WBC	Lymphocyte	-0.709	0.002
	Neutrophil	Lymphocyte	-0.809	<0.001
	Neutrophil	Myoglobin	-0.092	0.734
	Lymphocyte	Myoglobin	0.016	0.952

Linear regression analysis performed using the percentage change induced in each parameter by intense exercise. WBC represents white blood cell count.

## Discussion

As illustrated in Figure 1, in response to interval workouts completed on both the first and last days of the training camp, WBC count significantly increased in the P group even though there was no significant increase in the CT group, whereas neutrophil count increased and lymphocyte count decreased in both groups. In addition, as shown in Table 4, the relative percentage changes in WBC count accompanying exercise on both the first and last days of the training camp showed positive correlations with neutrophil counts and negative correlations with lymphocyte counts. Neutrophil and lymphocyte counts showed a negative correlation. In general, WBC count and neutrophil count are known to increase after intense exercise, while lymphocyte count is known to decrease, in athletes and healthy adults [14,21]. Among the WBCs that increased after intense exercise, neutrophils induce inflammation, which is believed to reduce lymphocyte count through pro-inflammatory cytokine and stress hormone production, which in turn causes a reduction in immunological function [22-25]. The above observations suggest that the interval exercise bouts performed on the first and last days of the training camp induced an inflammatory state, thus reducing immunological function. In addition, no significant increase in WBC count was observed in the CT group on the first day of the training camp and the increase in neutrophil count and reduction in lymphocyte count accompanying exercise were significantly suppressed compared to the P group. These results indicate that CT intake suppresses excessive increases in inflammatory reactions accompanying intense exercise, and thus suppresses the reduction of immunological function.

Through analysis using mice, CT was shown to increase the levels of GSH in organisms, as well as increasing humoral immune responses and increasing antigen-specific antibody production [4]. NAC, a precursor of GSH, was shown in clinical studies to significantly suppress the production of ROS from neutrophils increased through exercise [17-19]. These findings suggest that CT may suppress ROS production from neutrophils accumulated in skeletal muscles damaged through intense exercise, suppressing further accumulation of neutrophils and thus suppressing the excessive inflammatory reaction. Further, this suppression of excessive inflammatory reaction is believed to suppress the reduction of immunological function. To clarify these points, further analysis of GSH and ROS production from neutrophils in organisms during intense exercise as well as the effects of CT intake on oxidative stress are necessary.

In this study, it is suggested that CT intake suppressed excessive inflammatory reaction on the first day of the training camp and suppressed reduction of immunological function. However, on the last day of the training camp, other than a tendency for CT intake to suppress increased WBC and myoglobin following the interval training workout, no significant effect was observed in comparison to the P group. In this analysis, the extent of the relative changes in neutrophil and lymphocyte counts accompanying intense exercise decreased on the last day compared to the first day of the training camp. In fact, the percentage increase in neutrophil count in the P group on the first day of the training camp was 200.4 ± 6.9% (mean ± SEM), while that on the last day of the training camp, 149.5 ± 14.4%, was significantly lower (p = 0.015, paired *t*-test). The lymphocyte count dropped to 36.2 ± 4.3% and 56.8 ± 9.5% of pre-exercise values on the first and last days of the training camp, respectively, with lymphocyte reduction on the last day being slightly lower (p = 0.095, paired *t*-test). As shown in Figure 3C, a significant increase in salivary cortisol (and index of stress) was observed following intense exercise on the first day, but on the last day of the training camp (Figure 3D), no change was observed (P group; 245.7 ± 52.3 *vs. *100.2 ± 17.8%; p = 0.022, paired *t*-test). Relative changes in blood IL-6 level (indicator of inflammation) accompanying intense exercise tended to be lower on the last day compared to the first day of the training camp (P group; 514.4 ± 66.9 *vs. *406.3 ± 66.9%; p = 0.063, paired *t*-test). The above results indicated that no significant effect of CT intake was observed on the last day of the training camp because the subjects had developed stronger physical ability through continuous training during the training camp, and thus significant increases in inflammatory reaction or reduced immunological function did not occur to the same extent on the last day. Suzuki *et al. *reported that the percentage increase in neutrophil count accompanying exercise decreases with repeated training [24]. This suggests that CT intake may function to suppress excessive inflammatory reaction only when excessive inflammatory reaction occurs.

In this study, blood CPK and Mb levels were examined to study the breakdown of skeletal muscles accompanying intense exercise. As shown in Figure 2, both CPK and Mb levels increased significantly in both groups accompanying intense exercise on both the first and last days of the training camp. However, the percentage increase in Mb level following exercise was significantly lower in the CT group only on the first day of the training camp. CPK and Mb have both been reported to be discharged into blood by myocytolysis triggered by inflammation caused by intense exercise [14,26]. However, in this analysis, the percentage increase in CPK after exercise in the P group was 120-160%, while that in Mb was 800-950%. The increase in CPK after exercise has been reported to be late onset, while that in Mb level occurs immediately after exercising [24]. As the blood samples were collected immediately after exercise in this study, the CPK values measured here were probably not the peak value after exercise. As stated above, CT intake is thought to suppress excessive inflammatory reaction after intense exercise as well as attenuate reduced immunological function as we observed on the first day of the training camp. As shown in Table 4, the relative percentage change in Mb level accompanying intense exercise on the first day of the training camp tended to show a positive correlation with neutrophil count and had a significant negative correlation with lymphocyte count. As mentioned earlier, excessive inflammatory reaction in response to exercise has been reported to increase myocytolysis [14,24,26]. On the first day of the training camp, CT intake significantly suppressed the exercise-induced increase in blood Mb levels compared to P and, therefore, may have reduced myocytolysis. This was further associated with significantly less reduction in blood lymphocyte level compared to P. On the last day of the training camp, CT intake showed a tendency to suppress the increase in Mb level accompanying intense exercise when compared to the P group (Table 3). On the last day of the training camp, CT intake did not have any effect on neutrophil or lymphocyte counts, and linear regression analysis showed no correlations between the relative percentage change in Mb with either neutrophil or lymphocyte count. These results suggest that the suppression of Mb release caused by CT intake observed on the last day of the training camp is unrelated to inflammatory reactions, suggesting that CT may act directly on the skeletal muscles. On the other hand, the baseline value in CPK before the intense exercise on the last day of the camp was elevated compared with the first day as shown in Figure 2B. As mentioned above, the increase in CPK after exercise is late onset compared with that in Mb levels [24]. These findings suggest that the elevation of baseline CPK activity on the last day was due to the accumulation of daily intense exercise during the camp.

In this study, CT intake did not have any effect on the increase in salivary cortisol level accompanying intense exercise on the first day of the training camp. CT intake also did not have any effect on the increase in blood IL-6 level. IL-6, a pro-inflammatory cytokine, is known to promote secretion of cortisol through the hypothalamus-pituitary-adrenal axis [27,28]. In addition, IL-6 secretion accompanying intense exercise has been reported to be derived from skeletal muscle and not immune-competent cells [28,29]. Thus, CT intake is believed to have no effect on the increased IL-6 secretion from skeletal muscle accompanying intense exercise. As a result, there was no difference between the two groups in saliva cortisol levels. CT intake during intense exercise has the potential to suppress excessive inflammatory reactions as well as reduce immunological functions independent of increased pro-inflammatory cytokines derived from skeletal muscles. Further analyses by chronological sampling after exercise as well as measurement of pro-inflammatory cytokines other than IL-6 (IL-1, IL-8, and TNF) are necessary.

The proposed mechanism of action of CT during intense exercise is as follows. Intense exercise damages skeletal muscles, which are then infiltrated by neutrophils. The infiltrating neutrophils produce ROS, causing inflammatory reactions inside the skeletal muscles, thus destroying the tissues. This damage would further recruit neutrophils into the muscles causing excessive inflammatory reaction. Excessive inflammatory reaction reduces the lymphocyte count and lowers immunological function. In addition, excessive inflammatory reaction increases myocytolysis. The results of this study suggest that CT acts on neutrophils to suppress excessive inflammatory reactions, protect immunological function (blunted decline in lymphocytes), and reduce the breakdown of skeletal muscle caused by excessive inflammatory reactions. Thus, CT intake may contribute to the prevention of infectious disease in athletes during periods of intense training and suppress the breakdown of skeletal muscles through a mechanism involving the suppression of excessive inflammatory reaction.

## Conclusion

CT supplementation significantly inhibited the increase in neutrophil count and the reduction in lymphocyte count induced by intense endurance exercise. In addition, CT supplementation has a tendency to suppress the increase in Mb level induced by intense endurance exercise. These results suggest that CT supplementation may suppress the exercise-induced inflammatory response and may help to reduce immunosuppression and inflammatory-derived muscle damage in response to acute exercise stress.

Periods of increased training that commonly occur during training camps for athletes often are accompanied by high-intensity and high-frequency exercise that can lead to disturbance of the immune system. The present study supports other reports about CT supplementation that indicate the possibility that the consecutive intake of CT for at least 7 days before the camp suppresses the disturbance of immune function induced by high-intensity and high-frequency of exercise. Therefore, prophylactic supplementation with CT in persons training for high-intensity endurance exercise may, at least partially, support sustained immune function. Further research is needed to determine if CT supplementation can affect responses to chronic exercise stress and overtraining.

## Competing interests

This study was supported by Ajinomoto Co. Inc. The authors declare that they have no competing interests.

## Authors' contributions

SM assisted in recruitment of the participants, data collection, clinical supply management and drafting the manuscript. SK contributed to protocol development, statistical analysis and interpretation of the data and drafting the manuscript. CAT participated in supervision and provided oversight in drafting the manuscript. MO assisted in the study concept and manuscript preparation. All authors have read and approved the final manuscript.
